# Impact of instructional design on students’ critical thinking and engagement in online English reading classes: mediating role of motivation and moderating role of language proficiency

**DOI:** 10.3389/fpsyg.2025.1644126

**Published:** 2025-10-30

**Authors:** Qiuyang Huang, Mohd Mokhtar Muhamad, Nur Raihan Che Nawi

**Affiliations:** ^1^Jiangxi Arts and Ceramics Technology Institute, Jingdezhen, China; ^2^Universiti Putra Malaysia, Serdang, Malaysia

**Keywords:** critical thinking, instructional design, language proficiency, motivation, student engagement

## Abstract

This study investigates the association between instructional design, students’ critical thinking skills, and student engagement, as well as the mediating role of motivation and the moderating role of language proficiency. Data were collected from 380 students using a random sampling technique. A structured questionnaire was employed as the research instrument, and data were analyzed through structural equation modeling (SEM). The findings indicate that inquiry-based instructional design shows a significant positive relationship with both critical thinking skills and student engagement, whereas traditional instructional design shows a negative relationship. The blended instructional design demonstrates a positive relationship, though to a lesser extent than the inquiry-based approach. Moreover, motivation mediates the relationship between instructional design and both critical thinking skills and student engagement. Language proficiency moderates the relationship between instructional design and critical thinking skills, but not student engagement.

## 1 Introduction

Instructional design refers to the systematic process of creating effective and efficient learning experiences across educational, corporate training, and e-learning contexts (Kazemier et al., 2021). Effective instructional design integrates clear learning objectives, learner characteristics, learning environments, and available resources to support personalized and successful learning experiences (Rim and Shin, 2021).

Two essential components of academic achievement and personal development are critical thinking skills and student engagement. Critical thinking involves questioning assumptions, evaluating evidence, and considering multiple perspectives (Yeung et al., 2023). Student engagement, in contrast, reflects learners’ involvement and interest in the learning process (Lee and Ha, 2022). Engaged students actively participate in class, ask questions, and demonstrate persistence, which contributes to improved academic performance (Prifti, 2020).

Instructional design plays a significant role in fostering both critical thinking and student engagement. Well-structured educational designs can stimulate learners’ ability to analyze, evaluate, and synthesize information, thereby supporting informed decision-making (Panavas et al., 2022). Motivation is also a key factor in this relationship. Highly motivated students are more likely to actively participate in learning activities and develop critical thinking skills, while students with low motivation may disengage and fail to benefit from instructional strategies that promote higher-order thinking (Yeung et al., 2023). Similarly, language proficiency can influence students’ ability to fully engage in learning processes. Learners with higher language proficiency are better positioned to benefit from instructional approaches that encourage critical thinking, whereas those with limited language skills may face challenges in applying such strategies effectively.

Although previous research has explored instructional design, critical thinking, student engagement, motivation, and language proficiency in various contexts, notable gaps remain. Many studies have examined the effects of instructional design on critical thinking (e.g., Butz and Hancock, 2019), but relatively few have addressed the mediating role of motivation in linking instructional design to student engagement. Additionally, the moderating role of language proficiency in the relationship between instructional design, critical thinking, and engagement has received limited attention (Wang et al., 2020). Most existing studies focus on learning outcomes without fully considering how motivational and linguistic factors shape these dynamics.

To address these gaps, this study investigates the association between instructional design, critical thinking skills, and student engagement, with a particular focus on the mediating role of motivation and the moderating role of language proficiency. Specifically, this study focuses on the following core research questions:

1. What is the relationship between instructional design, students’ critical thinking skills, and student engagement?
2. How does motivation mediate the relationship between instructional design, critical thinking skills, and student engagement?
3. How does language proficiency moderate these relationships?

By examining these questions, the study aims to offer insights into how different instructional designs can be strategically used to enhance critical thinking and engagement, especially in linguistically diverse learning environments.

## 2 Literature review

### 2.1 Instructional design and cognitive load theory

Instructional design (ID) is a systematic process of developing educational experiences and materials to achieve specific learning objectives (Rojas and Benakli, 2020). Over the past decades, instructional design research has evolved from behaviorist models to constructivist and learner-centered approaches, reflecting shifts toward active and inquiry-based learning (Argelagós et al., 2022). Modern instructional design emphasizes personalized learning, blended learning environments, and the integration of technology such as gamification and learning analytics to enhance student engagement and outcomes (O’Rourke et al., 2021; Tatarinova et al., 2022).

Cognitive Load Theory (CLT) provides a critical theoretical lens for understanding the effectiveness of instructional design. According to CLT, instructional strategies should reduce extraneous cognitive load while enhancing germane cognitive load, enabling learners to process information more efficiently (Sweller, 2011). Inquiry-based instructional designs, for instance, have been shown to enhance critical thinking by encouraging active problem-solving and reflection, but their effectiveness depends on the careful management of cognitive demands (Zhang et al., 2021). Scaffolding strategies embedded within instructional design help students gradually build complex reasoning skills without overwhelming their cognitive capacity (Lantz-Andersson et al., 2022). Recent systematic reviews highlight that well-designed blended and inquiry-based instructional approaches consistently foster higher levels of engagement and critical thinking compared to traditional lecture-based methods, across diverse educational contexts (Maureen et al., 2022; Luo and Song, 2022; Huang et al., 2022).

### 2.2 Motivation and self-determination theory

Motivation is a key determinant of students’ learning behaviors and outcomes. Self-Determination Theory (SDT) offers a comprehensive framework for understanding motivation by distinguishing between intrinsic and extrinsic types and emphasizing the roles of autonomy, competence, and relatedness (Deci and Ryan, 2000). When students feel autonomous, competent, and connected to others, they are more likely to be intrinsically motivated and engaged in learning activities.

In the context of second language (L2) learning, Dörnyei’s (2001, 2010) L2 Motivational Self System expanded traditional integrative and instrumental motivation frameworks, proposing that learners’ “ideal L2 self” plays a crucial role in sustaining long-term motivation. Studies have demonstrated that teacher motivational practices, such as providing meaningful feedback, task variety, and supportive classroom environments, can significantly enhance learners’ intrinsic motivation and classroom engagement (Guilloteaux and Dörnyei, 2008).

However, compared to instructional design and language proficiency, the motivation literature is already extensive. Therefore, this review focuses on how motivation functions as a mediating mechanism linking instructional design with critical thinking and engagement, rather than detailing every motivational theory. Motivation determines how deeply students engage with instructional materials, influencing both cognitive outcomes (e.g., critical thinking) and behavioral outcomes (e.g., participation) (Berger-Estilita and Greif, 2020; Fondo and Gómez-Rey, 2021).

### 2.3 Language proficiency in instructional contexts

Language proficiency is a critical factor influencing students’ ability to comprehend, analyze, and engage with academic content, particularly in second language learning contexts. It encompasses learners’ capacity to use language accurately and appropriately in diverse contexts (Bardovi-Harlig et al., 2022). Proficiency development is affected by authentic language input, frequency of practice, quality of instruction, and learner motivation (Révész et al., 2022; Wang and Lee, 2021).

Recent research highlights the moderating role of language proficiency in shaping the relationship between instructional design, motivation, and learning outcomes. For example, Martí et al. (2020) and Belda-Medina (2022) found that students with higher language proficiency were more likely to benefit from inquiry-based instructional strategies that require critical analysis and synthesis of complex texts. Language proficiency facilitates comprehension of challenging materials and supports active participation in critical discussions (Din, 2020).

Cross-cultural studies have also shown that language proficiency moderates the relationship between motivation and critical thinking skills (Kong and Kang, 2020) as well as between motivation and engagement (Lee et al., 2022). For instance, students with higher proficiency levels exhibit stronger positive associations between motivation and engagement compared to those with lower proficiency (Aukerman and Chambers Schuldt, 2021). These findings suggest that language proficiency not only influences learning outcomes directly but also shapes how students respond to instructional designs and motivational factors across cultural contexts.

### 2.4 Critical thinking and student engagement

Critical thinking—the ability to evaluate information, challenge assumptions, and make reasoned judgments—is increasingly recognized as a core educational outcome (Qi, 2022; Yeung et al., 2023). Instructional designs that incorporate inquiry-based learning, problem-solving, and reflective activities have been consistently linked to the development of critical thinking skills (Liang, 2023; Zhang et al., 2021). Scaffolding and structured reflection further strengthen these skills by guiding students through complex cognitive processes (Rojas and Benakli, 2020).

Student engagement, encompassing behavioral, emotional, and cognitive dimensions, is another key determinant of academic success (Lee and Ha, 2022; Yang and Zhang, 2023). Engaged students participate actively, persist through challenges, and experience a sense of belonging in the learning community. Instructional designs that incorporate multimedia, gamification, or interactive learning environments have been shown to increase engagement across different educational settings (Huang et al., 2022; Luo and Song, 2022).

### 2.5 Integrative perspectives

Integrating these strands of literature highlights several key insights. First, instructional design, informed by Cognitive Load Theory, plays a central role in shaping students’ cognitive and behavioral engagement. Second, motivation, grounded in Self-Determination Theory, functions as a mediator that explains how instructional strategies translate into learning outcomes. Third, language proficiency serves as a moderator that can either amplify or constrain the effects of instructional design and motivation, particularly in linguistically diverse or L2 contexts.

Despite substantial research, gaps remain. Few studies examine the combined mediating role of motivation and moderating role of language proficiency in the relationship between instructional design, critical thinking, and engagement. Moreover, cross-cultural and multilingual perspectives are underrepresented in the literature. Addressing these gaps can deepen our understanding of how to design instructional strategies that are both cognitively effective and linguistically inclusive.

## 3 Hypothesis development and conceptual framework

The research objectives encompass a comprehensive investigation into the impact of instructional design on critical thinking skills and student engagement in online English reading classes, considering the mediating role of motivation and the moderating role of language proficiency. The objectives include empirically examining the influence of instructional design on critical thinking skills and student engagement, exploring its impact on student motivation, and assessing the reciprocal relationship between motivation and critical thinking skills as well as motivation and student engagement. The study also aims to scrutinize the mediating role of motivation in the connection between instructional design and critical thinking skills, and instructional design and student engagement. Furthermore, the research seeks to analyze how language proficiency moderates the relationships between motivation and critical thinking skills, and motivation and student engagement. These objectives collectively aim to provide a nuanced understanding of the complex dynamics among instructional design, motivation, language proficiency, student engagement, and critical thinking skills in the unique context of online English reading classes.

**Figure 1:**
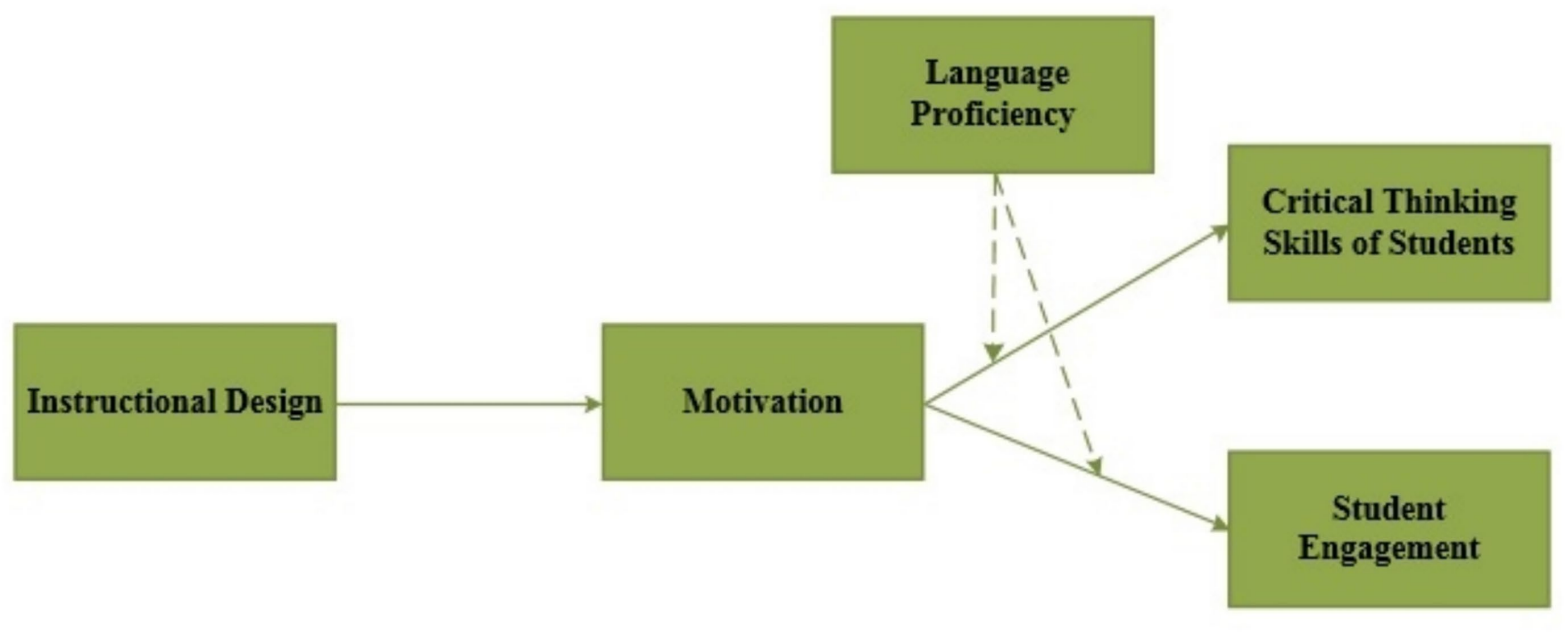
Conceptual framework.

On the basis of our literature review we purpose that ID has a Significant and positive impact on CTSS, SE, and M. furthermore motivation also has significant and CTSS and SE. M mediates the relationship between CTSS and SE on the other hand LP moderates the relationship between M and CTSS and SE. thus on the basis of this we develop the following hypothesis and the conceptual framework shown in Figure 1.

*H1*: Instructional design has a significant and positive impact on critical thinking skills of students.

*H2*: Instructional design has a significant and positive impact on student engagement.

*H3*: Instructional design has a significant and positive impact on motivation.

*H4*: Motivation has a significant and positive impact on critical thinking of students.

*H5*: Motivation has a significant and positive impact on student engagement.

*H6*: Motivation mediates the relationship between instructional design and critical thinking skills of student.

*H7*: Motivation mediates the relationship between instructional design and student engagement.

*H8*: Language proficiency moderates the relationship between motivation and critical thinking skills of students.

*H9*: Language proficiency moderates the relationship between motivation and student engagement.

In order to improve readability, researchers have included important acronyms in this study that are intended to reinforce and simplify important ideas:

ID (Design for Instruction): The term “instructional design” describes the online English reading course’s structure and instructional framework. Throughout the study, instructional design plays a crucial role in developing critical thinking abilities, facilitating student learning, and enhancing engagement through the creative design of educational activities and resources.

CTSS (Critical Thinking Skills of Students): Students’ critical thinking abilities are reflected in CTSS. In order to determine the possible influence of instructional design on academic success, we investigate in our research how students’ critical thinking abilities in online English reading courses are influenced by instructional design.

SE (Student Engagement): The level of involvement students show during learning activities is referred to as student engagement. In an effort to gain a better knowledge of the online learning environment, we look into how instructional design influences student engagement.

M (Motivation): Our study, which focuses on the influence of student learning motivation in online English reading courses, uses motivation as a mediating element. We want to reveal how instructional design indirectly improves students’ critical thinking abilities and engagement by impacting motivation. Regarding motivation, including both pre-class and in-class motivation.

LP (Language Proficiency): An indicator of students’ degrees of language competency, language proficiency is a moderating factor. We explore the moderating role of language proficiency in the impact of instructional design on students’ critical thinking skills and engagement, aiming to understand performance variations among students with different language proficiencies.

## 4 Methodology

### 4.1 Research design

This study employed a correlational research design to examine the relationships among instructional design, critical thinking skills, student engagement, motivation, and language proficiency. The research was conducted in two phases: questionnaire development and pilot testing, followed by large-scale questionnaire administration.

### 4.2 Participants and sampling

A total of 380 undergraduate students participated in the study. Participants were recruited through random sampling between May 30 and June 15, 2023. The gender distribution was approximately balanced, with 48% male and 52% female participants, ensuring representativeness and minimizing gender-related bias. All participants were English as a Second Language (ESL) learners with diverse proficiency levels, verified through standardized tests (e.g., TOEFL, IELTS) and self-assessment.

### 4.3 Instruments

A structured self-administered questionnaire was developed to measure the core variables of the study. Instructional design was assessed through items evaluating students’ perceptions of inquiry-based, traditional, and blended approaches (e.g., “The course activities encouraged me to think critically about the reading materials”). Critical thinking skills were measured using adapted items from validated critical thinking scales, such as “I can identify underlying assumptions in the reading texts.” Student engagement was examined through items targeting cognitive and behavioral aspects (e.g., “I actively participate in online discussions”). Motivation was measured using items adapted from established motivation scales, focusing on learning goals and persistence (e.g., “I set clear goals for my English reading improvement”). Language proficiency was evaluated through self-assessment items covering vocabulary mastery, comprehension ability, and the use of learning strategies (e.g., “I can understand academic English texts without frequent dictionary use”). All items were rated on a 5-point Likert scale (1 = strongly disagree, 5 = strongly agree). Prior to the main data collection, the questionnaire was pilot-tested with 30 students to ensure clarity and reliability. Cronbach’s alpha values for each scale exceeded 0.80, indicating good internal consistency.

### 4.4 Data collection

Data were collected through online questionnaires. Participants provided informed consent before participation. Anonymity and confidentiality were strictly maintained. The data collection period lasted approximately 2 weeks.

### 4.5 Data analysis

Data were analyzed using SPSS and the PROCESS macro following a series of systematic procedures. First, descriptive statistics were conducted to summarize the demographic characteristics of the participants and to examine the distributions of the key variables. Next, exploratory factor analysis (EFA) was performed to validate the underlying structure of the questionnaire and to confirm construct validity. Following this, correlation analysis was carried out to explore the bivariate relationships among instructional design, critical thinking skills, student engagement, motivation, and language proficiency. Subsequently, regression analyses were employed to test the associations between these variables and to determine the strength and direction of the relationships. Finally, mediation and moderation analyses were conducted using PROCESS Model 4 (for mediation) and Model 1 (for moderation) to examine the mediating role of motivation and the moderating role of language proficiency in the relationships between instructional design, critical thinking skills, and student engagement.

## 5 Results

### 5.1 Exploratory factor analysis

An exploratory factor analysis (EFA) was conducted to examine the underlying structure of the questionnaire. The KMO measure was 0.857, indicating sampling adequacy for factor analysis. The Bartlett’s test of sphericity determines whether or not the correlation matrix of the variables is an identity matrix (meaning that the variables do not have any correlation with one another) or whether or not there are significant correlations among the variables, which is a prerequisite for factor analysis. A preliminary chi-square statistic, degrees of freedom (df), and probability value (*p*-value) are generated by the test (sig.). In this instance, the chi-square value is around 5234.527, the number of degrees of freedom is 180, and the *p*-value is 0.000. Table 1 shows the result of KMO and Bartlett’s Test.

**Table 1:** KMO and Bartlett’s test.

KMO and Bartlett’s test		
Kaiser–Meyer–Olkin measure of sampling adequacy.		0.857
Bartlett’s test of sphericity	Approx. Chi-Square	5234.527
	Df	180
	Sig.	**0.000**

Bold values indicate key test statistics: the Kaiser-Meyer-Olkin (KMO) measure of sampling adequacy, the chi-square value of Bartlett’s Test of Sphericity, and its significance level (*p*-value).

The approach known as major component analysis was utilized in order to extract components and common factors from the 25 items, which resulted in the production of the initial factor load matrix for the questionnaire. After that, in accordance with the oblique rotation method, the loading matrix of the rotated factors was obtained. Table 2 shows the result of EFA analysis.

**Table 2:** Exploratory factor analysis.

Item	Instructional design	Critical thinking skills of student	Student engagement	Motivation	Language proficiency	Communalities
ID1	0.669					0.741
ID2	0.699					0.669
ID3	0.574					0.737
ID4	0.614					0.699
ID5	0.612					0.608
CTSS1		0.842				0.869
CTSS2		0.857				0.887
CTSS3		0.842				0.853
CTSS4		0.804				0.902
CTSS5		0.799				0.885
SE1			0.843			0.871
SE2			0.859			0.889
SE3			0.841			0.843
SE4			0.809			0.904
SE5			0.799			0.885
M1				0.741		0.797
M2				0.744		0.803
M3				0.715		0.785
M4				0.706		0.937
M5				0.769		0.733
LP1					0.754	0.558
LP2					0.636	0.776
LP3					0.502	0.738
LP4					0.595	0.757
LP5					0.527	0.811

After conducting the EFA, we calculate the value of Cronbach alpha for each variable in order to check the reliability of the questionnaire. Cronbach’s alpha is a measure of internal consistency reliability, which indicates how well a set of items or questions measures a single construct or concept. Cronbach’s alpha coefficients of 0.7 or higher are generally regarded as indicating good internal consistency. All of the variables have strong internal consistency based on this criterion, with Cronbach’s alpha coefficients ranging from 0.799 for Linguistic Proficiency to 0.934 for Student Engagement. This implies that the items used to measure each of these factors are extremely trustworthy and consistent. Table 3 displays the results of the reliability analysis.

**Table 3:** Reliability analysis.

Variables	Cronbach alpha
Instructional design	0.874
Critical thinking skills of student	0.927
Student engagement	0.934
Motivation	0.904
Language proficiency	0.799

The Pearson correlation coefficient (−1 to +1) measures the linear relationship between two variables. A correlation of 1 is perfect, 0 is no correlation, and −1 is perfect negative. Instructional design favorably and marginally affects student critical thinking, engagement, motivation, and language proficiency. Critical Thinking Skills of Student is highly correlated with Student Engagement and strongly correlated with Motivation. Student Engagement is highly correlated with Motivation. Motivation is positively and moderately correlated with Language Proficiency. Finally, Language Proficiency is positively and weakly correlated with Instructional Design, Critical Thinking Skills of Student, Student Engagement, and Motivation. Table 4 shows the result of correlational analysis.

**Table 4:** Correlation matrix.

Variables		Instructional design	Critical thinking skills of student	Student engagement	Motivation	Language proficiency
Instructional design	Pearson correlation	1	0.611^**^	0.615^**^	0.555^**^	0.281^**^
	Sig. (1-tailed)		0.000	0.000	0.000	0.000
	*N*	380	380	380	380	380
Critical thinking skills of student	Pearson Correlation	0.611^**^	1	0.999^**^	0.659^**^	0.442^**^
	Sig. (1-tailed)	0.000		0.000	0.000	0.000
	*N*	380	380	380	380	380
Student engagement	Pearson Correlation	0.615^**^	0.999^**^	1	0.662^**^	0.441^**^
	Sig. (1-tailed)	0.000	0.000		0.000	0.000
	*N*	380	380	380	380	380
Motivation	Pearson Correlation	0.555^**^	0.659^**^	0.662^**^	1	0.664^**^
	Sig. (1-tailed)	0.000	0.000	0.000		0.000
	*N*	380	380	380	380	380
Language proficiency	Pearson Correlation	0.281^**^	0.442^**^	0.441^**^	0.664^**^	1
	Sig. (1-tailed)	0.000	0.000	0.000	0.000	
	*N*	380	380	380	380	380

**Correlation is significant at the 0.01 level (1-tailed).

The hypothesis was tested using beta, R2, *F*-value, and *p*-value. The beta value for each independent variable reflects the direction and strength of the association between them. The independent variable explains *R*^2^ of the dependent variable’s variance. The *F*-value measures model significance. Finally, the p-value indicates the probability of obtaining the observed results by chance. Findings show that ID has significant and positive impact on CTSS, SE and M (*p* < 0.05). Furthermore, M also has significant and positive impact on CTSS and SE (*p* < 0.05). Table 5 shows the result of regression analysis. Table 5 shows the result of regression analysis.

**Table 5:** Regression analysis.

Hypothesis	Relation	Beta value	*R* ^2^	*F*	*P*-value	Hypothesis Supported
H1	ID → CTSS	0.420	0.373	224.969	0.000	Yes
H2	ID → SE	0.327	0.387	229.539	0.000	Yes
H3	ID → M	0.555	0.555	167.910	0.000	Yes
H4	M → CTSS	0.609	0.659	290.520	0.000	Yes
H5	M → SE	0.613	0.662	294.739	0.000	Yes

ID, instructional design; CTSS, critical thinking skills of student; SE, student engagement; M, motivation; LP, language proficiency.

Coefficients indicate individual indirect effects and standard errors for various levels of conscientiousness, as well as the bottom and upper limits of 95% BC bootstrap confidence intervals for that effect, calculated using 1,000 bootstrap samples. Low values are one standard deviation below the mean, mean values are one standard deviation above the mean, and high values are one standard deviation above the mean. IND stands for indirect effects; LLCI stands for lower level of confidence interval; and ULCI stands for upper level of confidence interval. Table 6 shows the result of indirect effect.

**Table 6:** Mediating analysis.

Hypothesis	Relation	IND	SE	LLCI	ULCI	Hypothesis supported
H6	ID → M → CTSS	0.139	0.031	0.078	0.202	Yes
H7	ID → M → SE	0.134	0.319	0.074	0.199	Yes

ID, instructional design; CTSS, critical thinking skills of student; SE, student engagement; M, motivation.

Table 7 presents the results of a statistical analysis, where two hypotheses (H8 and H9) are tested for moderating effect of LP between the relationship of M, CTSS and SE. For both hypotheses, the beta coefficients are positive (0.1977 for H8 and 0.1984 for H9), indicating a positive relationship between M x LP and CTSS and SE, respectively. The standard errors (SE) are relatively small, suggesting a high level of precision in the estimates. The *T*-values for both hypotheses are large (8.4301 for H8 and 8.3826 for H9), which means that the coefficients are significant at a high level of confidence (*p* < 0.001). The *p*-values are both equal to 0.0000, indicating that the probability of observing such results by chance alone is extremely low. Therefore, both hypotheses are supported by the data.

**Table 7:** Moderation effect.

Hypothesis	Relation	Beta	SE	T value	*p*-value	Hypothesis supported
H8	M x LP → CTSS	0.1977	0.0235	8.4301	0.0000	Yes
H9	M x LP → SE	0.1984	0.0237	8.3826	0.0000	Yes

CTSS, critical thinking skills of student; SE, student engagement; M, motivation; LP, language proficiency.

## 6 Discussion

The findings of this study reveal several significant relationships among instructional design, critical thinking skills, student engagement, motivation, and language proficiency. Instructional design shows a significant positive association with students’ critical thinking skills and engagement, as well-structured strategies provide clear guidance and promote active learning. Motivation mediates the relationships between instructional design and both critical thinking skills and engagement, indicating that when students perceive instructional activities as meaningful and autonomous, they are more motivated to participate actively, which in turn fosters higher levels of analytical and reflective abilities. Language proficiency moderates the relationship between motivation and both critical thinking skills and engagement, with higher-proficiency students benefiting more from motivational factors, while lower-proficiency students may struggle to fully engage with cognitively demanding tasks. These results underscore the interconnected roles of instructional design, motivation, and language proficiency in shaping students’ learning experiences and highlight the importance of integrating motivational strategies and differentiated language support to create more inclusive and effective online learning environments.

### 6.1 Discussion of H1

The first hypothesis is to investigate the instructional design has a significant and positive impact on critical thinking skills of students. Instructional design is a vital component of the teaching and learning process that must be used effectively for students to have good educational experiences. One key area where it has been discovered that instructional design affects students’ capacity to develop critical thinking abilities. Research has repeatedly demonstrated that instructional design significantly and favorably affects students’ acquisition of critical thinking abilities. A research by Sinaga et al. (2022), for instance, discovered that students who received instruction intended to foster critical thinking showed noticeably improved skills in knowledge analysis, evaluation, and synthesis. Another study by Panavas et al. (2022) discovered that instructional design approaches that include metacognitive and reflective practices can help students develop their critical thinking abilities. In online learning contexts, where students have the same amount of face-to-face engagement with their instructors and peers, the use of instructional design to encourage critical thinking abilities can be especially beneficial. In order to effectively foster critical thinking abilities among online learners, a study by Huang et al. (2022) found that problem-based learning, case-based learning, and collaborative learning may all be incorporated into online instructional design.

### 6.2 Discussion of H2

Second hypothesis states that instructional design has a significant and positive impact on student engagement. The systematic process of creating effective and efficient instructional materials that encourage learning is known as instructional design (Luo and Song, 2022). It entails assessing the needs of the learners, developing instructional strategies, and assessing the efficacy of the created products. Enhancing student engagement, which is described as “the level of interest and involvement that a learner demonstrates in their learning” (Al Mamun et al., 2020), is one of the key objectives of instructional design. First and foremost, instructional design aids in producing educational resources that are pertinent and significant to learners. Designing tools that encourage deep learning and lessen cognitive burden is crucial. By examining the needs, objectives, and prior knowledge of learners and developing resources that are suited to their learning preferences, instructional designers can accomplish this (Huang et al., 2022). Learners are more likely to interact with the resources and meet their learning goals if they do this. Second, active learning is encouraged by instructional design, and research shows that this increases student engagement (Lapitan et al., 2023). By encouraging students to participate in conversations, group projects, and problem-solving exercises, active learning engages students in the learning process (Hurwitz, 2023). To boost student engagement, instructional designers might create materials that combine active learning techniques including simulations, case studies, and peer-to-peer interaction.

### 6.3 Discussion of H3

According to the third hypothesis, motivation is significantly and positively influenced by instructional design. Instructional design refers to the systematic approach of developing educational and training resources that are characterized by their efficacy, efficiency, and engagement. According to Johnson et al. (2019), empirical evidence indicates that the implementation of instructional design can yield a noteworthy and favorable influence on learner motivation. Enhancing the pertinence of the subject matter being taught is a crucial means by which instructional design can influence motivation. According to Montag et al. (2021), learners’ motivation to engage with material is enhanced when they perceive its relevance to their goals and interests. The attainment of this objective can be facilitated by employing strategies such as establishing specific targets and furnishing practical illustrations and implementations of the subject matter. Instructional design can influence motivation by affording learners with chances for autonomy and self-direction. According to Reeve et al. (2022), learners are more inclined to feel motivated to interact with the material when they possess a sense of control over their learning. The implementation of strategies such as offering alternatives in tasks and exercises, as well as granting learners the autonomy to establish their own educational objectives, can facilitate the attainment of this outcome. The utilization of instructional design can potentially augment self-efficacy beliefs by employing strategies such as furnishing feedback that accentuates progress and accomplishment and implementing scaffolding techniques to gradually elevate the complexity of learning activities.

### 6.4 Discussion of H4

According to the fourth hypothesis, there exists a positive and significant correlation between motivation and critical thinking abilities among students. Motivation is an essential component of the learning process that propels students toward attaining academic excellence. The findings of Loi and Thanh (2022) study indicate that motivation exerts a noteworthy and favorable influence on the critical thinking abilities of students. Initially, research has indicated a robust association between motivation and critical thinking. According to Tang et al. (2020), critical thinking was significantly predicted by motivation. According to the study, students who exhibited high levels of motivation demonstrated superior performance on critical thinking assignments in comparison to their less motivated counterparts. The study found that highly motivated pupils performed better on critical thinking assignments. Motivating pupils to be curious and excited about learning can also improve critical thinking. According to Batini et al. (2021), motivation plays a crucial role in fostering a favorable disposition toward learning, resulting in increased levels of involvement and active engagement. Consequently, this fosters the enhancement of cognitive abilities related to analytical reasoning. In addition, the enhancement of motivation can facilitate students’ capacity to self-manage their learning, a fundamental aspect for the cultivation of analytical reasoning abilities. Akcaoğlu et al. (2023) conducted a study which found that the relationship between motivation and critical thinking is mediated by self-regulation. The study revealed that students who displayed high levels of motivation exhibited superior aptitude in regulating their learning behavior and employing metacognitive strategies to augment their critical thinking abilities.

### 6.5 Discussion of H5

The fifth hypothesis asserts that student involvement is significantly and favorably impacted by motivation. Students’ engagement in their studies is significantly influenced by their motivation. Motivated students are more engaged in class, which improves academic performance and learning attitudes (Lee et al., 2022). Extrinsic and intrinsic motivation exist. Extrinsic motivation originates from prizes, grades, or praise, while intrinsic motivation derives from personal interest or delight in the activity (Li et al., 2021). Intrinsic motivation predicts engagement and academic success more than extrinsic (Leung and Law, 2019). Motivated pupils think critically and analyze. Questioning presumptions, assessing the facts, and reaching well-informed conclusions are all parts of engaging students. According to Guo et al. (2023), students who are driven to study are more likely to participate in critical thinking and analysis, which results in deeper learning and improved academic achievement.

### 6.6 Discussion of H6

Hypothesis six posits that the relationship between instructional design and critical thinking skills of students is significantly mediated by motivation. The inquiry into the correlation between instructional design and critical thinking abilities has garnered attention in the realm of educational scholarship (Cheng et al., 2023). Recent research has demonstrated that motivation significantly mediates this relationship, demonstrating that the effectiveness of instructional design in fostering critical thinking abilities depends on how motivated students are to engage in that process (e.g., Cheon et al., 2019; Dong et al., 2021). According to Wong (2020), instructional designs that include motivating components, such as opportunities for autonomy and feedback, can boost students’ interest in learning and encourage them to use critical thinking.

### 6.7 Discussion of H7

According to the seventh hypothesis, there exists a significant mediating effect of Motivation on the association between instructional design and student engagement. According to Darfler and Kalantari (2022), the likelihood of students engaging with instructional materials and engaging in critical thinking is higher when they are motivated to learn. When students lack motivation, they may disengage from learning, which lowers critical thinking skills (Atabek et al., 2022). The relationship between instructional design and motivation has been discovered to be interconnected by research, and motivation has been shown to play a substantial mediating role in the connection between instructional design and student engagement (Lapitan et al., 2023). To clarify, the impact of instructional design on fostering critical student engagement is contingent upon the degree of student motivation. According to Froment and De-Besa Gutiérrez (2022), students who exhibit motivation may demonstrate a greater propensity to actively engage in classroom discourse, pose inquiries, and undertake autonomous research. The aforementioned actions have the potential to foster the development of analytical skills and augment the level of involvement of pupils.

### 6.8 Discussion of H8

The eighth hypothesis states that students’ linguistic competence significantly moderates the link between motivation and critical thinking. Teng et al. (2020) claim that students’ language competency and motivation significantly influence their development of critical thinking skills. Recent studies show that language proficiency affects motivation and critical thinking. Language proficiency is essential for analytical reasoning development. Bağ and Gürsoy (2021), assert that language proficiency plays a pivotal role in the attainment of critical thinking abilities. The research conducted by Li (2022) has revealed that motivation is a noteworthy predictor of critical thinking abilities among students. The correlation between motivation and critical thinking skills is more pronounced among students who possess advanced language proficiency, in contrast to those who exhibit lower levels of language proficiency. According to Puspita and Amelia (2020) findings, the correlation between motivation and critical thinking abilities is notably more pronounced among students who possess proficient language skills.

### 6.9 Discussion of H9

According to the ninth hypothesis, there exists a significant moderation effect of language proficiency on the association between motivation and student engagement. The correlation between motivation and student engagement is significantly influenced by language proficiency (Müller-Brauers et al., 2020). The role of motivation in fostering student engagement is widely acknowledged in academic literature. Empirical studies have established a positive correlation between motivation and academic achievement, as well as favorable learning outcomes (Flunger et al., 2022). Li et al. (2022) highlight how students’ linguistic competence affects their drive and interest in learning a second language. Advanced language learners are more likely to participate in language learning activities since they can understand and communicate better. Gan (2020) believes that low-proficient language learners may struggle to participate in language learning activities due to their lack of confidence and the difficulty of the problems they face.

In conclusion, instructional design plays a crucial role in students’ critical thinking skills, enhancing knowledge analysis, evaluation, and synthesis. Online learning contexts can benefit from instructional design, incorporating problem-based learning, case-based learning, and collaborative learning. Design also influences student engagement by creating effective learning materials, encouraging deep learning, and reducing cognitive burden. Motivation is a key factor in students’ critical thinking abilities, with high motivation leading to superior performance and better regulation of learning behavior. Intrinsic motivation, derived from personal interest, predicts engagement and academic success more than extrinsic motivation. Motivation mediates the relationship between instructional design and student engagement, and language proficiency significantly influences the relationship between motivation and critical thinking. Advanced language learners are more likely to participate in language learning activities, while low-proficient learners may struggle. Overall, instructional design plays a significant role in fostering critical thinking and student engagement.

## 7 Implications

### 7.1 Theoretical implications

The study underscores the significance of instructional design in fostering the enhancement of students’ critical thinking abilities and their engagement in the educational process. The statement underscores the importance of educators and instructional designers in creating and executing effective instructional designs that foster critical thinking and student engagement. These designs can be developed and implemented by educators and instructional designers. According to the study’s findings, motivation is a significant moderator in the relationship between pedagogical method and students’ critical thinking abilities and level of engagement in the learning process. This underscores the significance of instructional designers and educators integrating motivational techniques into the instructional designs they develop for their pupils. The study’s results indicate that linguistic competence plays a crucial role as a moderating factor in the correlation between instructional design, critical thinking abilities, and student engagement. The statement suggests that educators ought to carefully design instructional materials by considering the present linguistic proficiency of their students prior to delivering the lessons to the entire class.

### 7.2 Practical implications

This study emphasizes the importance of well-structured course design in online English reading classes. It encourages educators to enhance course structure by incorporating interactive content and developing critical thinking skills. Educators should also cultivate students’ motivation through strategies like embedding motivational elements, providing constructive feedback, and creating a supportive online environment. This approach not only influences knowledge transmission but also emotional and motivational aspects.

Tailoring interventions based on individual students’ language abilities is crucial for creating an inclusive learning environment. Instructors should provide additional support, resources, or task complexity based on proficiency. Continuous professional development is essential for online educators to design and deliver courses that address critical thinking, motivation, and language-related factors.

The study suggests continuous assessment and feedback mechanisms, incorporating formative tools for real-time feedback, and fostering constructive peer feedback to enhance critical thinking skills and motivation in online English reading classes, thereby improving student engagement.

### 7.3 Limitations and future directions

#### 7.3.1 Limitations

It is probable that the results of this study cannot be applied to all student populations because the sample may not be representative of all linguistic proficiency levels. The absence of a control group in the study poses a challenge in establishing causality and attributing the enhancements in critical thinking skills solely to the instructional design. The reason for this can be attributed solely to the modification of the instructional design. The utilization of self-reporting measures as the sole means of assessing participants’ motivation levels in the study raises the possibility that the outcomes may not furnish a comprehensive depiction of the interplay between instructional design and critical thinking skills concerning motivation. The cross-sectional design of the study poses a challenge in assessing the progression of critical thinking skills among participants over time.

#### 7.3.2 Future directions

Employing a longitudinal study methodology may prove advantageous in acquiring a more profound comprehension of the correlation between instructional design, motivation levels, and critical thinking proficiencies over an extended period. In forthcoming times, research endeavors may employ diverse methodologies to assess individuals’ degrees of motivation, including observational or physiological measures. The potential for gaining a more precise comprehension of the moderating impact of language proficiency on the association between instructional design and critical thinking skills could be enhanced by incorporating students with diverse levels of linguistic competence in the research. Subsequent research endeavors could entail a comparative analysis of the effectiveness of diverse pedagogical approaches in fostering students’ critical thinking proficiencies and levels of engagement. Possible academic rewrite: A potential area for future research is the interplay among instructional design, motivation, and critical thinking skills, with a focus on contextual factors such as cultural diversity, curriculum content, and pedagogical approaches. Such investigations could shed light on how these elements influence each other.

## 8 Conclusion

This study highlights the significant associations between instructional design, students’ critical thinking skills, and student engagement, with motivation acting as a mediating factor and language proficiency serving as a moderating factor. The findings suggest that well-structured instructional designs are closely related to higher levels of student engagement and the development of critical thinking skills. Motivation emerges as a pivotal element in strengthening these relationships, while language proficiency influences the extent to which students can fully benefit from instructional strategies.

Academically, this study contributes to the growing body of literature by providing a more nuanced understanding of how instructional design, motivation, and language proficiency interact in shaping students’ learning experiences. It moves beyond examining isolated effects and instead explores their interconnected relationships within the specific context of online English reading classes. Practically, the results offer valuable implications for educators and instructional designers, emphasizing the importance of integrating motivational elements and language support into instructional planning to foster more inclusive and effective learning environments.

While these findings provide meaningful insights, the study’s reliance on self-reported, cross-sectional data and its focus on a single learning context limit the generalizability of the results. Future research should adopt longitudinal or mixed-method approaches and extend the scope to different subjects and educational contexts to further validate and expand upon these findings.

## Data Availability

The original contributions presented in the study are included in the article/supplementary material, further inquiries can be directed to the corresponding author.
